# Elective Tracheal Intubation With the VieScope—A Prospective Randomized Non-inferiority Pilot Study (VieScOP-Trial)

**DOI:** 10.3389/fmed.2022.820847

**Published:** 2022-03-15

**Authors:** Martin Petzoldt, Yasmin Engels, Zohal Popal, Pischtaz A. Tariparast, Phillip B. Sasu, Andrés Brockmann, Mark A. Punke, Jörn Grensemann

**Affiliations:** ^1^Department of Anesthesiology, Center of Anesthesiology and Intensive Care Medicine, University Medical-Center Hamburg-Eppendorf, Hamburg, Germany; ^2^Department of Intensive Care Medicine, Center of Anesthesiology and Intensive Care Medicine, University Medical-Center Hamburg-Eppendorf, Hamburg, Germany

**Keywords:** airway management, VieScope, intubation, laryngoscopy, laryngoscope, bougie

## Abstract

**Background:**

Tracheal intubation is commonly performed after direct laryngoscopy using Macintosh laryngoscopes (MacL), but visualization of the larynx may be inadequate. The VieScope (VSC) as a new type of laryngoscope consisting of a straight, shielded, illuminated tube used to perform intubation *via* a bougie was investigated in this prospective randomized trial in patients without expected difficult airways.

**Methods:**

With ethics approval, 2 × 29 patients for elective surgery were randomized 1:1 to intubation with VSC or MacL. Endpoints were first attempt success rates (FAS), Percentage of Glottis Opening Scale (POGO), time to intubation (TTI), and difficulty ratings on visual analog scales (0–100, lower values better). Data are given as mean ± standard deviation.

**Results:**

The FAS was 83 ± 38% for VSC and 86 ± 34% for MacL (*P* = 0.723). For VSC, POGO was 86 ± 17% and for MacL 68 ± 30% (*P* = 0.007). TTI for VSC was 93 ± 67s vs. 38 ± 17 for MacL (*P* < 0.001). Difficulty of intubation was rated 23 ± 22 for VSC vs. 18 ± 22 for MacL (*P* = 0.422), viewing conditions 12 ± 15 vs. 24 ± 25 (*P* = 0.031), and difficulty of tube placement was rated 27 ± 30 vs. 7 ± 8 (*P* = 0.001).

**Conclusion:**

No difference in FAS was detected between VSC and MacL. Visualization of the larynx was superior using the VSC, while TTI was prolonged and tube placement *via* bougie was more challenging. The VSC could be an alternative to MacL in patients with difficult laryngoscopy, but this should be investigated further in patients with expected difficult airways.

## Introduction

Tracheal intubation is required for mechanical ventilation and to prevent pulmonary aspiration in patients in the operating room, intensive care unit, and in emergency medicine. The clinical standard method for tracheal intubation is direct laryngoscopy (DL) with Macintosh blades (1, 2). However, this technique has some limitations and difficulties and may fail due to insufficient visualization of the larynx or due to difficult advancement of the tracheal tube through the laryngeal inlet (3, 4). A new device has been introduced that consists of an illuminated straight closed circular tube with a beveled end for laryngoscopy (VSC, Vie Scope, Adroit Surgical, Oklahoma City, OK, USA) that enables for intubation facilitated by a bougie. With the VSC approach the epiglottis is elevated which is the typical maneuver for the so called “straight blade technique” (5). As opposed to other straight blade devices as, i.e., the Miller laryngoscope, the visual axis is shielded due to the circular tube design and thus protected from secretions. The VSC has been inspired by suspension laryngoscopy used for microlaryngoscopy and laryngeal surgery in otorhinolaryngology for many decades. It has been claimed that the VSC might provide superior visualization of the larynx as compared to DL with Macintosh laryngoscopes.

Feasibility of the VSC in normal and difficult airways has been evaluated in manikin studies with promising results (6, 7) and the VSC has been shown to be superior to conventional DL during simulated cardiopulmonary resuscitation with providers wearing personal protective equipment (8). However, clinical studies comparing the VSC with DL as the current clinical standard technique are missing, so far.

Therefore, we aimed to study the VSC in patients undergoing elective surgery with a predicted non-difficult airway vs. conventional DL in a prospective randomized pilot trial. We hypothesized that the VSC was non-inferior to conventional DL with Macintosh laryngoscopes concerning first attempt success rates and visualization.

## Methods

### Ethics

The study was approved by the Ethics Committee of the Hamburg Chamber of Physicians (2020-10238-BO-ff, December 21, 2020, chairman Prof. Dr. Stahl). All patients provided written informed consent. The study was registered prior to patient enrollment on ClinicalTrials.gov (NCT04724408, submitted for registration: January 23, 2021) and conducted in accordance with the Declaration of Helsinki and adheres to the applicable CONSORT guidelines.

### Trial Design

The VieScOP trial was a prospective randomized non-inferiority investigator-initiated pilot study with a 1:1 allocation ratio to either VSC or conventional DL with Macintosh laryngoscopes.

### Eligibility

The trial was conducted in the Center of Anesthesiology and Intensive Care Medicine at the University Medical Center, Hamburg-Eppendorf. Patients were eligible if they were at least 18 years old, required transoral tracheal intubation for elective otorhinolaryngologic or oral and maxillofacial surgery, and had no evidence of a difficult airway. To evaluate airway difficulty, a structured preoperative airway assessment was conducted according to in-house standards that included a physical examination with respect to anatomical conditions associated with difficult intubation, i. e. a restricted mobility of the cervical spine, retrognathia, and obesity, thyromental distance, and mouth opening. Mallampati Scores (9) and the Simplified Airway Risk Index (SARI) were obtained (10, 11). Patients with an indication for nasotracheal intubation, special tubes as e. g. laser or RAE tubes, rapid-sequence induction, and loose teeth were excluded.

### Interventions

In patients randomized to the intervention group tracheal intubation was attempted by means of the VSC. After visualization of the larynx, a bougie with a straight tip (Tactical Bougie, Adroit Surgical, Oklahoma City, OK, USA) was introduced into the trachea, and the VSC withdrawn. In a second step, the tracheal tube was placed over this bougie for tracheal intubation. In patients randomized to the control group tracheal intubation was attempted with a conventional Macintosh type laryngoscopy in a single stage approach.

The approach to visualization with the VSC (midline or paraglossal), the choice of the blade and tube size, as well as the use of adjuncts like stylets, introducers or forceps was unrestricted and was at the discretion of the attending anesthetist. To facilitate visualization of the larynx, airway optimization maneuvers [e.g., backward upward rightward pressure (BURP) and optimum external laryngeal manipulation (OELM)] could be employed. Anesthesia induction was performed with propofol, and sufentanil or remifentanil. For neuromuscular blocking, either rocuronium or mivacurium were used.

### Outcome Parameters

The primary outcome measure was the first attempt success rate defined as intubation with only one laryngoscopy, intubation, and for the VSC one bougie placement attempt until successful tracheal intubation. Secondary outcome parameters were the Percentage of Glottis Opening Scale (POGO) (12), the Cormack-Lehane grade (13), the overall success rate, time to successful intubation, time to successful intubation with one attempt, end-tidal carbon dioxide concentration following intubation, total number of attempts to successful intubation, and average number of attempts for intubation. The difficulty of glottis visualization, difficulty of tube placement, and the overall difficulty of intubation were rated on visual analog scales (0–100, lower values better). Time to successful intubation was measured from the laryngoscope passing the teeth to the first of at least three positive end-tidal carbon dioxide readings without significant visual decrease in capnography (side stream capnography, Primus Anesthesia Workstation, Drägerwerk AG, Lübeck, Germany). After a maximum of three intubation attempts with any device the method was recorded as unsuccessful and further intubation attempts had to be commenced with a different technique, i.e., videolaryngoscopy. Any retraction of the laryngoscope, tube or bougie was defined as an additional attempt. All complications were recorded, particularly regurgitation or aspiration during intubation, accidental esophageal intubation, signs of hypoxemia defined as a decrease of the pulse oximetric saturation below 80%, and hypotension as a decrease of the systolic blood pressure below 70 mmHg (Infinity Delta vital signs monitor, Drägerwerk AG, Lübeck, Germany).

### Participating Physicians

All participating anesthetists were trained with the VSC in a structured manikin airway training to avoid a bias due to an inferior skill level for this device. It has been previously shown that paramedics with little clinical experience may reliably intubate a manikin after 30 min of lecture followed by 10 min of familiarization with the VSC (6). Participating anesthetists received a structured 30 min VSC familiarization training before participating in this study. The duration of professional experience of the participating anesthetists was recorded.

### Sample Size Analysis

According to an *a priori* sample size calculation, 2 × 29 patients were required with errors of α = 0.025 and β = 0.2 to show non-inferiority for the intervention method, based on a first attempt success rate of 40% (14), and a non-inferiority margin of 5% (PASS version 08.0.6, NCSS, LLC. Kaysville, UT, USA).

### Randomization

Sealed, opaque envelopes were used for randomization. Envelopes containing the randomization code to either intubation with the VSC or with Macintosh laryngoscopes were opened after the anesthetist was assigned to the patient.

### Statistics

Microsoft Excel 2016 (Microsoft Corp., Redmond, WA, USA) was used for data management and the SPSS statistical software package (version 25, IBM Inc., Armonk, NY, USA) was used for statistical analysis. We used *t*-tests for comparisons of parameters as well as contingency tables with Chi-square and Fisher's tests. Two-tailed *P*-values < 0.05 were regarded as statistically significant.

## Results

**Figure 1:**
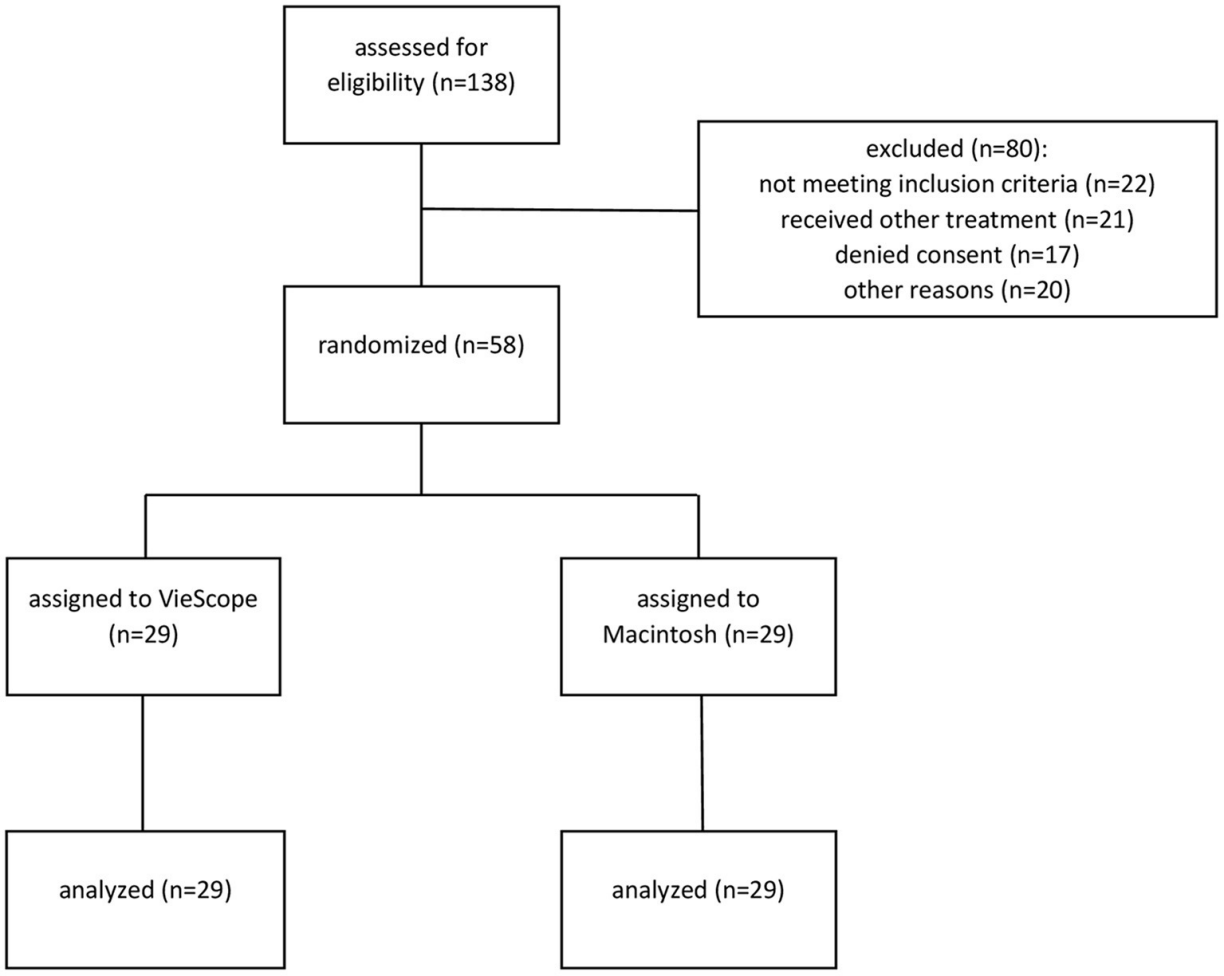
Consolidated standards of reporting (CONSORT) diagram.

From January 25, 2021 to March 1, 2021, 58 patients receiving tracheal intubation were randomized to either intubation with the VSC or with a Macintosh laryngoscope in a 1:1 ratio (Figure 1). Patients' baseline characteristics are shown in Table 1. All patients randomized to VSC were intubated *via* the midline approach.

**Table 1:** Patient characteristics.

**Parameter**	**VieScope** **(*n* = 29)**	**Macintosh** **(*n* = 29)**
Age (years)	49 ± 20	58 ± 16
Sex	Male: *n* = 14 (48%)	Male: *n* = 19 (66%)
	Female: *n* = 15 (52%)	Female: *n* = 10 (34%)
Height (m)	1.74 ± 0.10	1.76 ± 0.10
Weight (kg)	81 ± 24	80 ± 20
ASA	1: *n* = 10 (34%)	1: *n* = 7 (24%)
	2: *n* = 11 (38%)	2: *n* = 19 (66%)
	3: *n* = 8 (28%)	3: *n* = 3 (10%)
SARI (points)	1.2 ± 1.1	1.3 ± 1.4
Mouth opening (cm)	>4: *n* = 27 (93%)	>4: *n* = 26 (90%)
	4: *n* = 2 (7%)	4: *n* = 3 (10%)
	<4: *n* = 0	<4: *n* = 0
Thyromental distance (cm)	>6.5: *n* = 26 (90%)	>6.5: *n* = 23 (79%)
	6–6.5: *n* = 3 (10%)	6–6.5: *n* = 6 (21%)
	<6: *n* = 0	<6: *n* = 0
Mallampati-Score	1: *n* = 5 (17%)	1: n=8 (28%)
	2: *n* = 14 (48%)	2: *n* = 11 (38%)
	3: *n* = 10 (34%)	3: *n* = 9 (31%)
	4: *n* = 0	4: *n* = 1 (3%)

*ASA, Physical Status Classification System according to the American Society of Anesthesiologists; SARI, Simplified Airway Risk Index. Data are shown as mean ± standard deviation*.

**Figure 2:**
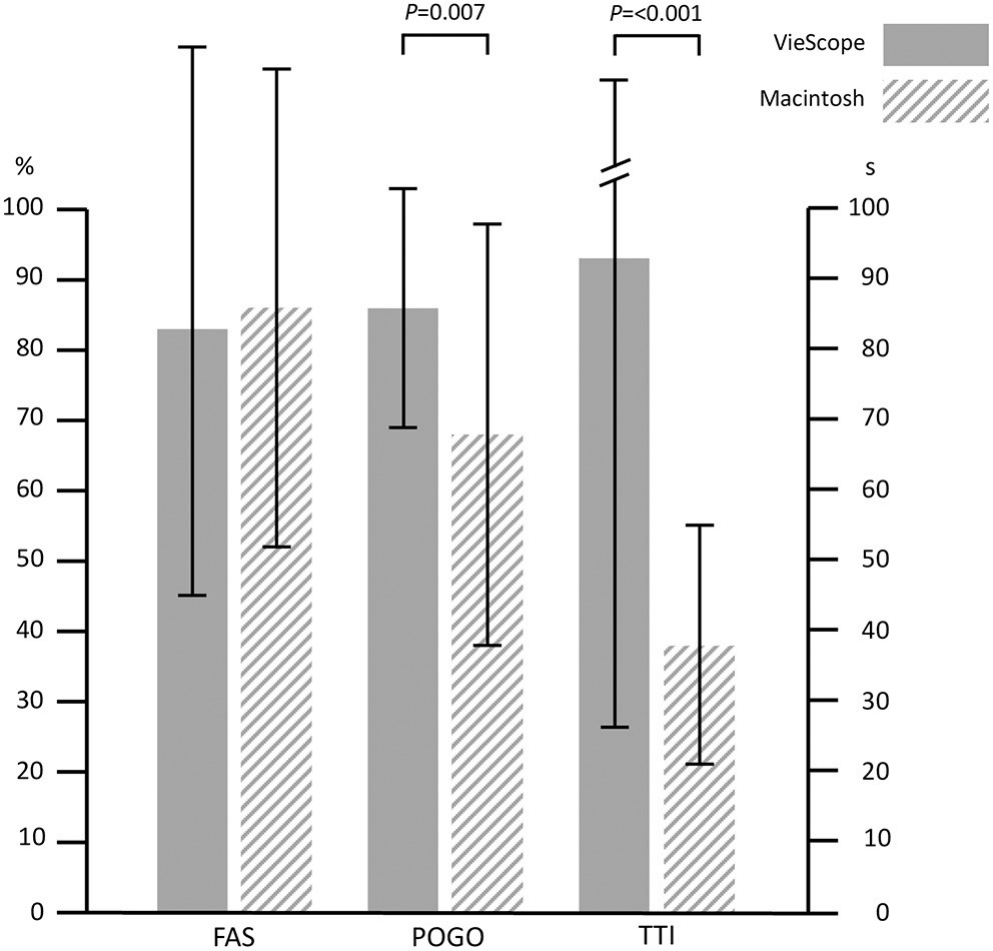
First attempt success rates, visualization, and time to intubation. FAS, First attempt success rate; POGO, Percentage of Glottis Opening Scale; TTI, Time to intubation; Error bars indicate standard deviation.

**Figure 3:**
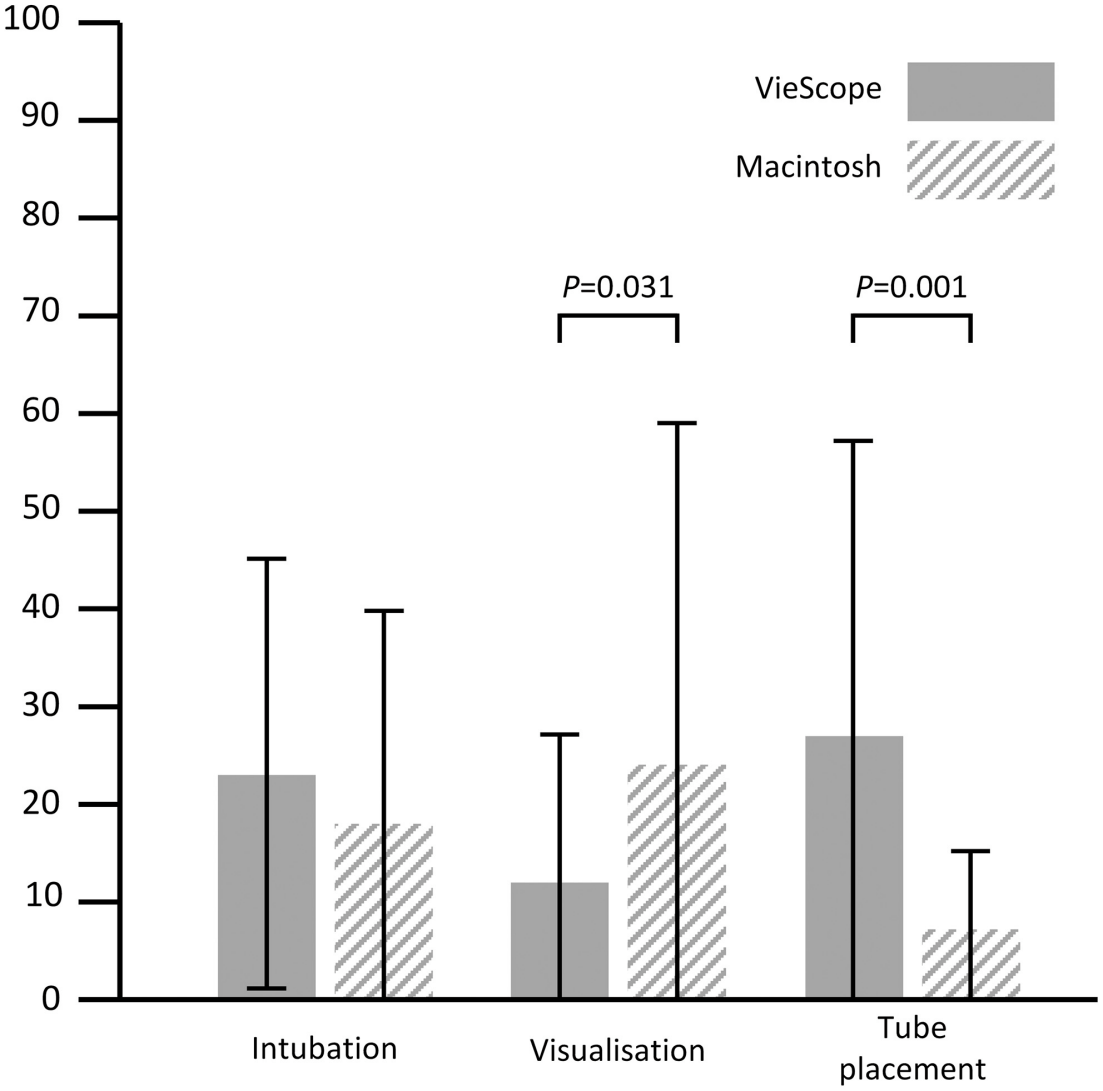
Intubation difficulty ratings on visual analog scales. All items rated on visual analog scales (0–100, lower values better); intubation: overall difficulty of intubation; visualization: difficulty to obtain adequate view on larynx; Tube placement: difficulty of advancing tube into trachea; Error bars indicate standard deviation.

The first attempt success rate was 83% in the VSC group and 86% in the Macintosh group with a mean difference of −3% (95% confidence intervals −23 to 16%), *P* = 0.723. The overall success rate was 100% in both groups. POGO was superior in the VSC group with 86 ± 17% vs. 68 ± 30%, *P* = 0.007. Time to intubation was prolonged in the VSC group with 93 ± 67 s vs. 38 ± 17 s, *P* < 0.001. An overview on results is given in Table 2 and Figures 2, 3. All participating anesthetists were specialists with a mean professional experience of 18 ± 4 years in the VSC group vs. 19 ± 4 years in the control group (*P* = 0.781). The reasons for multiple attempts are given in Supplementary Table S1. No difference in the patient characteristics could be found between first attempt success or multiple attempts (Supplementary Table S2). Maneuvers facilitating intubation are given in Supplementary Table S3.

**Table 2:** Outcome parameters.

**Parameter**	**VieScope** **(*n* = 29)**	**Macintosh** **(*n* = 29)**	***p***
First-attempt success rate (%)	83%	86%	0.723
POGO (%)	86% ± 17	68% ± 30	0.007
Cormack/Lehane grade	1: *n* = 19 (66%)	1: *n* = 12 (41%)	0.012
	2: *n* = 10 (34%)	2: *n* = 17 (59%)	
Average number of laryngoscopic attempts	1.2 ± 0.5	1.0 ± 0.2	0.146
Number of laryngoscopic attempts	1: *n* = 25 (86%)	1: *n* = 28 (96%)	0.338
	2: *n* = 3 (10%)	2: *n* = 1 (3%)	
	3: *n* = 1 (3%)		
Average number of intubation attempts	1 ± 0.5	1 ± 0.3	0.511
Number of intubation attempts	1: *n* = 25 (86 %)	1: *n* = 26 (90%)	0.601
	2: *n* = 3 (10%)	2: *n* = 3 (10%)	
	3: *n* = 1 (3%)		
Time to successful intubation (s)	93 ± 67	38 ± 17	<0.001
Time to successful intubation with one attempt (s)	74 ± 30	33 ± 8	<0.001
Time to successful bougie placement (s)	49 ± 68	n/a	n/a
SpO_2_ after intubation (%)	99 ± 1	99 ± 1	0.445
etCO_2_ after intubation (mmHg)	33 ± 5	31 ± 5	0.145
Regurgitation/aspiration during intubation	none	none	n/a
Accidental esophageal intubation	*n* = 1 (3%)	none	0.322
SpO_2_ <80%	none	none	
Systolic blood pressure <70 mmHg	*n* = 1 (3%)	*n* = 2 (7%)	0.561
Difficulty intubation (VAS)	23 ± 22	18 ± 22	0.422
Difficulty view conditions (VAS)	12 ± 15	24 ± 25	0.031
Difficulty tube placement (VAS)	27 ± 30	7 ± 8	0.001

*SpO_2_, pulse oximetric saturation; POGO, Percentage of Glottis Opening Scale; VAS, rated on visual analog scales (0–100, lower values better). Data are shown as mean ± standard deviation; n/a, not applicable*.

## Discussion

In this prospective randomized trial, we did not find a difference between tracheal intubation with the VSC and the Macintosh laryngoscopes in patients without an expected difficult airway regarding the first attempt success rate. Interestingly, visualization of the larynx as assessed by POGO and Cormack/Lehane scores was superior in the VSC group. On the other hand, intubation with the VSC required significantly more time than the conventional approach.

So far, the VSC has only been studied in simulated airway scenarios (6–8). Although helpful in the initial assessment of new devices, manikin studies have limitations as, e.g., secretions obscuring the view on the larynx are often not adequately modeled (15).

One of the important parameters assessing new intubation techniques is the first attempt success rate because more than two attempts have been associated with severe complications and even death (16). The achieved rates depend on the experience of the intubating physician and one study could show first attempt success rates from 72% for physicians at the first postgraduate year to 89% for consultant anesthetists with an overall average of 83% (17). The same average rate of 83% was found in a meta-analysis including nearly 1,000 patients (18, 19). All intubations in our study were conducted by experienced fellow or consultant anesthetists and the first pass success rates of both devices are comparable to the published data. The slightly lower first pass success rates observed may be attributable to the patients from the otorhinolaryngologic, and oral- and maxillofacial surgery departments with a higher incidence of difficult airways, although patients with predicted difficult airways were excluded (20, 21). Furthermore, only cases in which laryngoscopy, bougie placement (if applicable), and tube advancement were successful at the first attempt were counted in favor of the first attempt success rate.

Essentially, the VSC is a type of straight blade laryngoscope. For Miller blade laryngoscopes as another straight blade type, an incidence of difficult intubation has been shown to be ~5% in adult patients (22) while 9% have been reported for the Macintosh blade (23). The latter study by Arino et al. evaluating different laryngoscope blade styles concluded that curved blades increase the ease of intubation while straight blades improve the visualization of the larynx (23). Two studies evaluating the conventional Macintosh technique vs. a paraglossal and retromolar Miller blade approach found an improved laryngeal view using the Miller blade (24, 25). Intubation with the VSC as a straight laryngoscope supports this finding providing a superior view on the larynx as shown by an increased POGO score, and lower Cormack-Lehane and visualization ratings on the visual analog scales in our study, although all patients were intubated *via* the midline approach. On the other hand, a study conducted in an emergency medical services setting could show a first attempt success rate for Macintosh blades of 86 vs. 73% for Miller laryngoscopes but no information was provided on the skill level of the intubating paramedics (26). As the intubation technique with straight blades or the VSC differs from traditional curved blade laryngoscopes, we deem proper training as mandatory before using straight blade devices and we therefore introduced a structured manikin training for the participating anesthetists in our study to ensure that a sufficient learning curve was reached before the first patient was attended.

The overall difficulty of intubation rated on visual analog scales did not differ between Macintosh and VSC. While glottis visualization was rated better, tube placement was rated poorer in the VSC cohort. Inherent to the VSC approach, the laryngeal inlet is not visualized during tube placement and tracheal tube impingement on the arytenoid cartilages is a known problem with bougie facilitated intubation that can usually be resolved by a 90° tube rotation (27, 28). However, this maneuver and the bougie facilitated intubation itself are time-consuming. For our control group, the time to successful intubation was in accordance with previously published data (15). For the VSC, time to successful intubation was far longer. Although this did not have a relevant influence on oxygen saturation or carbon dioxide accumulation it may be of importance in pre-hospital or critically ill patients with acute respiratory dysfunction and impaired oxygenation (29). Interestingly, an improved visualization of the laryngeal inlet with an increased time to intubation has been shown recently for a different device for intubation, as well (30).

The tube design necessitates a two-step approach with intubation over a bougie as the endotracheal tube may not be passed through the VSC tube itself. However, the bougie may also be advantageous as insertion into the trachea may even be possible in cases of inferior visualization. A recent randomized study with more than 750 patients conducted in an emergency department found an improved first attempt success rate for intubation over a bougie of 96 vs. 83% for conventional Macintosh intubation (28).

Complications from elective anesthesia in patients without a predicted difficult airway are rare events with aspiration estimated at ~1:4,000 (31). Therefore, no aspiration was likely to occur in this study. For accidental esophageal intubation, an incidence of 1:25 and 1:250 has been reported for direct laryngoscopy (28, 32) with the difference being attributable to different degrees of anesthetists' experience. The one esophageal intubation in the VSC group presumably occurred by chance.

Our study has certain limitations. By study design, it was not possible to blind the anesthetist from the used device leading to a risk of observer bias. Being the first study with a new device the effect size for our study could not be approximated a priory due to a paucity of existing data. Thus, this pilot study has an exploratory character. As one of the major aims of this pilot study was to obtain data for sample sizes approximations for future confirmatory studies, we used a pragmatic approach for the sample size calculation by choosing a low first-pass-success rate for the VSC as demonstrated for other devices in intensive care. While inferiority could have been shown with this approach, our study may have been underpowered to show non-inferiority. All participating anesthetists were specialists with at least 10 years of experience. The handling of the VSC and especially the paraglossal approach may be counterintuitive for those who predominantly intubate using Macintosh laryngoscopes and it is unclear whether 30 min of manikin training with the VSC were sufficient. All intubations with the VSC occurred *via* the midline approach and it is unknown if the paraglossal approach could have increased the high first attempt success rate even further. The marketing focus for this device is the prehospital environment with providers not as well trained as the participating staff anesthetists in this study and the results of this study may not be transferable to emergency medical services providers with fewer airway training.

## Conclusions

In patients requiring elective tracheal intubation for surgery without a predicted difficult airway, the first attempt success rate of the VSC was comparable to conventional laryngoscopy with curved Macintosh type blades. The VSC provided superior visualization of the larynx while the time to intubation was prolonged due to the bougie facilitated intubation. In the future, the VSC should be evaluated in other settings as, e.g., in intensive care or pre-hospital patients, and in patients with a predicted difficult airway to define patient groups benefitting from intubation with the VSC.

## Data Availability Statement

The raw data supporting the conclusions of this article will be made available by the authors, without undue reservation.

## Ethics Statement

The studies involving human participants were reviewed and approved by Ethics Committee of the Hamburg Chamber of Physicians (2020-10238-BO-ff, December 21, 2020, Chairman Prof. Dr. Stahl). The patients/participants provided their written informed consent to participate in this study.

## Author Contributions

MPe designed the study, helped to write the manuscript, interpreted the data, and supervised the study conduct in the operating room. YE obtained the data, recruited patients, and helped with the statistical analysis. MPu, ZP, and PS contributed to the study conduct and revised the manuscript. PT and AB helped with the statistical analysis and revised the manuscript. JG designed the study, wrote the manuscript, performed the statistical analysis, and helped to interpret the data. All authors contributed to the article and approved the submitted version.

## Funding

Expenses of this investigator-initiated trial were covered from departmental funds except the VieScope laryngoscopes that were kindly provided by Adroit Surgical (Oklahoma City, OK, USA) free of charge.

## Conflict of Interest

MPe received a research grant awarded by Verathon. PT and AB have received study support from Ambu. JG has received study support from ETView, Ambu, Pfizer, and Infectopharm, and received consultant and lecture fees from Drägerwerk, GE Healthcare, Fresenius Medical, and Smith Medical. The remaining authors declare that the research was conducted in the absence of any commercial or financial relationships that could be construed as a potential conflict of interest.

## Publisher's Note

All claims expressed in this article are solely those of the authors and do not necessarily represent those of their affiliated organizations, or those of the publisher, the editors and the reviewers. Any product that may be evaluated in this article, or claim that may be made by its manufacturer, is not guaranteed or endorsed by the publisher.
