# Supplementary material for: Elective Tracheal Intubation With the VieScope—A Prospective Randomized Non-inferiority Pilot Study (VieScOP-Trial)
Source: Front Med (Lausanne). 2022 Mar 15;9:820847. doi: 10.3389/fmed.2022.820847 (PMC8964792; doi:10.3389/fmed.2022.820847)
Supplement: Supplementary file 1 [file Table_1.DOCX]

# Table S1: Reasons for multiple attempts

VieScope (5 patients)

- repositioning of patient
- two attempts for bougie advancement required
- accidental esophageal intubation
- tube diameter too large
- suspected tracheal stenosis (tube could not be advanced at first attempt)

Macintosh (4 patients)

- technical defect of laryngoscope (inadequate illumination)
- difficult visualization, need for stylet
- difficult visualization, need for stylet and repositioning of patient
- tube could not be advanced at first attempt

**Table S2:** Patients’ characteristics depending on first attempt success rate

| **Parameter** | **first attempt success (n=49)** | **multiple attempts (n=9)** | **p** |
| --- | --- | --- | --- |
| **Age (years)** | 54 ± 18 | 50 ± 21 | 0.555 |
| **Sex** | Male: n=27  Female: n=6 | Male: n=6  Female: n=3 | 0.520 |
| **Height (m)** | 1.74 ± 0.10 | 1.79 ± 0.10 | 0.200 |
| **Weight (kg)** | 80 ± 21 | 83 ± 26 | 0.720 |
| **ASA** | 1: n=14  2: n=25  3: n=10 | 1: n=3  2: n=5  3: n=1 | 0.436 |
| **SARI (points)** | 1.3 ± 1.3 | 1.2 ± 1.1 | 0.889 |
| **Mouth opening (cm)** | >4: n=45  4: n=4  <4: n=0 | >4: n=8  4: n=1  <4: n=0 | 0.772 |
| **Thyromental distance (cm)** | >6.5: n=40  6-6.5: n=9  <6: n=0 | >6.5: n=9  6-6.5: n=0  <6: n=0 | 0.162 |
| **Mallampati-score** | 1: n=10  2: n=22  3: n=16  4: n=1 | 1: n=3  2: n=3  3: n=3  4: n=0 | 0.987 |

ASA: Physical Status Classification System according to the American Society of Anesthesiologists; SARI: Simplified Airway Risk Index; Data are shown as mean ± standard deviation.

**Table S3:** Maneuvers facilitating intubation

| **Parameter** | **VieScope (n=29)** | **Macintosh (n=29)** | **p** |
| --- | --- | --- | --- |
| **Airway optimization maneuvers (BURP/OELM)** | n= 4 | n=7 | 0.315 |
| **Repositioning of patient** | n=1 | n=1 | 1.000 |
| **Laryngoscope or blade change** | n=0 | n=0 | n/a |
| **Tube change** | n=1 | n=0 | 0.313 |
| **Stylet** | n/a | n=2 | n/a |
| **Magill forceps** | n=0 | n=0 | n/a |

BURP: backward upward rightward pressure; OELM: optimum external laryngeal manipulation; n/a: not applicable.
